# Early-Onset Severe Preeclampsia as an Independent Predictor of New-Onset Maternal Arrhythmia During Pregnancy: A Retrospective Cohort Study

**DOI:** 10.7759/cureus.107550

**Published:** 2026-04-22

**Authors:** Teddy A Teddy, Spencer Cadet, Abdelwahab Ahmed, Fraol T Erega, Mustafa Marzoug, Nicole C Sparling, Kendall Bell

**Affiliations:** 1 Internal Medicine, Detroit Medical Center/Wayne State University, Detroit, USA; 2 Internal Medicine, HCA/University of Central Florida (UCF) Fort Walton Beach Hospital, Fort Walton Beach, USA; 3 Medical School, University of Louisville School of Medicine, Louisville, USA; 4 Cardiology, Wayne State University, Detroit, USA

**Keywords:** cardio-obstetrics, cardiovascular complications, early-onset preeclampsia, endothelial dysfunction, maternal arrhythmia, preeclampsia, pregnancy, propensity score matching, retrospective cohort study

## Abstract

Background

Early-onset severe preeclampsia (EOSPE) is characterized by marked vascular injury, systemic inflammation, and acute hemodynamic stress. However, its independent association with newly occurring maternal arrhythmias during pregnancy has not been well established.

Objective

To evaluate whether EOSPE independently increases the risk of new-onset maternal arrhythmia using propensity score matching.

Methods

We conducted a retrospective cohort study using the TriNetX database from 2015 to 2024. Pregnant individuals aged 18 to 45 years were included. EOSPE was defined as preeclampsia with severe features occurring before 34 weeks of gestation according to American College of Obstetricians and Gynecologists criteria. The primary outcome was new-onset maternal arrhythmia, including supraventricular tachycardia, atrial fibrillation or flutter, ventricular arrhythmias, clinically significant ectopy, or conduction disorders occurring during pregnancy or within six weeks postpartum. Patients with prior arrhythmia or structural heart disease were excluded. After applying inclusion and exclusion criteria, 742 EOSPE patients were matched 1:1 with controls without hypertensive disorders of pregnancy. Matching variables included age, race, body mass index, chronic hypertension, diabetes mellitus, chronic kidney disease, obesity, tobacco use, and prior adverse pregnancy history. Statistical analyses included conditional logistic regression, multivariable adjustment, Cox proportional hazards modeling, and subgroup interaction testing.

Results

Following matching, baseline characteristics were well balanced with standardized mean differences below 0.08. New-onset maternal arrhythmia occurred in 6.2% (46/742) of EOSPE patients compared with 2.4% (18/742) of controls, corresponding to an absolute risk difference of 3.8%. EOSPE was associated with increased odds of arrhythmia (odds ratio 2.68, 95% confidence interval 1.53 to 4.70; p < 0.001), which remained significant after adjustment (adjusted odds ratio 2.49, 95% confidence interval 1.39 to 4.45; p = 0.002). Time-to-event analysis demonstrated a higher hazard of arrhythmia (hazard ratio 2.41, 95% confidence interval 1.38 to 4.20; log-rank p < 0.001). Subtype analysis showed increased rates of supraventricular tachycardia (3.5% vs. 1.3%) and atrial fibrillation or flutter (1.5% vs. 0.4%) in the EOSPE group.

Conclusions

EOSPE is independently associated with a significantly increased risk of new-onset maternal arrhythmia during pregnancy and the postpartum period. These findings support closer cardiovascular monitoring in this high-risk population.

## Introduction

Preeclampsia affects approximately 5% to 8% of pregnancies worldwide and remains a leading cause of maternal and perinatal morbidity and mortality [1]. Among hypertensive disorders of pregnancy (HDP), early-onset severe preeclampsia (EOSPE), defined by the presence of severe features before 34 weeks of gestation, represents a high-risk phenotype characterized by placental dysfunction, systemic endothelial injury, and multiorgan involvement [2]. In addition to acute complications such as eclampsia, pulmonary edema, and acute kidney injury, EOSPE is associated with an increased long-term risk of cardiovascular disease (CVD), including hypertension, ischemic heart disease, stroke, and heart failure [3].

Although preeclampsia is increasingly recognized as a cardiovascular risk state, most prior studies have focused on long-term outcomes occurring years after pregnancy. Far less attention has been directed toward acute cardiovascular complications during the index pregnancy, particularly cardiac arrhythmias [4]. This gap is clinically important, as arrhythmias during pregnancy may lead to hemodynamic instability, thromboembolic events, and adverse maternal and fetal outcomes.

Cardiac rhythm disturbances are increasingly identified during pregnancy, with supraventricular tachycardia (SVT) representing the most common sustained arrhythmia. Physiologic adaptations of pregnancy, including increased plasma volume, elevated resting heart rate, reduced systemic vascular resistance, and enhanced sympathetic activity, create a substrate that may promote arrhythmogenesis [5]. However, most available data have focused on women with pre-existing cardiac conditions such as congenital heart disease (CHD), cardiomyopathy, or inherited arrhythmia syndromes. The extent to which new arrhythmias develop in previously healthy women with pregnancy-specific conditions such as EOSPE remains unclear.

Existing evidence linking preeclampsia to maternal arrhythmia is limited and largely derived from small observational studies or case series. These studies often lack appropriate control groups and do not adequately adjust for confounding factors such as chronic hypertension (CHTN), obesity, and diabetes mellitus (DM), all of which independently increase arrhythmia risk. Consequently, it is uncertain whether any observed association reflects shared baseline risk factors or a direct effect of the preeclamptic state itself.

Several biologically plausible mechanisms support a potential link between EOSPE and arrhythmia. Severe endothelial dysfunction and systemic inflammation are associated with increased circulating antiangiogenic factors and oxidative stress, which may alter cardiac ion channel function and promote electrical instability. Acute elevations in blood pressure and afterload can impair left ventricular (LV) relaxation and increase left atrial (LA) pressure, creating a substrate for atrial arrhythmias. Increased sympathetic activity and catecholamine release may further facilitate abnormal electrical activity. In addition, electrolyte disturbances such as hypokalemia and hypomagnesemia, which may occur in this population, are known contributors to arrhythmia development [5,6].

Given these considerations, there is a need for large-scale studies that rigorously evaluate the relationship between EOSPE and new-onset maternal arrhythmia. Therefore, we conducted a multi-institutional retrospective cohort study using the TriNetX research network to determine whether EOSPE independently predicts the development of maternal arrhythmias during pregnancy and the early postpartum period. To strengthen causal inference, we applied propensity score matching (PSM), multivariable adjustment, and time-to-event analysis.

## Materials and methods

This study utilized the TriNetX research network, a federated database providing access to de-identified electronic health record (EHR) data from over 120 million patients across more than 70 healthcare organizations, including academic medical centers, community hospitals, and specialty clinics [7]. The database includes demographic information, diagnostic codes based on the International Classification of Diseases, Tenth Revision (ICD-10), procedural data, laboratory values, medication records, and vital signs. Because all data were de-identified and aggregated prior to analysis, this study was exempt from institutional review board approval, and the requirement for informed consent was waived.

We conducted a retrospective cohort study including pregnant patients aged 18 to 45 years between January 1, 2015, and December 31, 2024. Pregnancy episodes were identified using ICD-10 codes for antenatal care, delivery, or pregnancy-related complications. The exposure of interest was EOSPE, defined according to American College of Obstetricians and Gynecologists criteria as preeclampsia with severe features occurring before 34 weeks of gestation. Severe features included systolic blood pressure of at least 160 mmHg or diastolic blood pressure of at least 110 mmHg on two occasions, thrombocytopenia with platelet count below 100,000 per microliter, renal dysfunction with serum creatinine greater than 1.1 mg per dL or doubling of baseline, hepatic injury with aspartate aminotransferase (AST) or alanine aminotransferase (ALT) at least twice the upper limit of normal, pulmonary edema, or new-onset neurologic or visual symptoms [1].

The control group consisted of pregnant patients without HDP during the index pregnancy. The primary outcome was new-onset maternal arrhythmia, defined as the first occurrence of any of the following during pregnancy or within six weeks postpartum: SVT, atrial fibrillation (AF) or atrial flutter, ventricular arrhythmias, premature atrial or ventricular contractions requiring clinical evaluation, or clinically significant conduction disorders [8]. To ensure incident cases, patients with any arrhythmia diagnosis in the 12 months prior to pregnancy were excluded.

Inclusion criteria required singleton pregnancy, documented gestational age, and follow-up through six weeks postpartum. Exclusion criteria included prior arrhythmia, CHD, cardiomyopathy, ischemic heart disease, inherited arrhythmia syndromes, clinically significant valvular heart disease, prior cardiac surgery, prior myocardial infarction, multiple gestation, advanced CKD with estimated glomerular filtration rate below 30 mL per minute per 1.73 m², type 1 DM with end-organ complications, and substance use disorder other than tobacco [9].

Baseline covariates were obtained from the 12 months preceding pregnancy and included maternal age, race and ethnicity, body mass index (BMI), obesity defined as BMI of at least 30 kg per m², CHTN, DM, CKD, thyroid disease, autoimmune disease, tobacco use, and prior adverse pregnancy history [2]. Additional variables included baseline heart rate, systolic and diastolic blood pressure, serum creatinine, platelet count, AST, ALT, family history of CVD, and baseline medication use.

To reduce confounding, we performed 1:1 PSM. The propensity score was estimated using logistic regression including all baseline covariates. Matching was conducted using a nearest-neighbor algorithm without replacement with a caliper width of 0.1 pooled standard deviations [10,11]. Covariate balance was assessed using standardized mean differences (SMD), with values below 0.10 indicating adequate balance [12].

All statistical analyses were performed using the TriNetX platform and validated using STATA version 18.0 (StataCorp, College Station, TX, USA). In the matched cohort, the primary analysis compared the incidence of new-onset maternal arrhythmia between EOSPE and control groups. Odds ratios (OR) with 95% confidence intervals (CI) were calculated using conditional logistic regression. Relative risk (RR) and absolute risk difference (ARD) were also calculated. Statistical significance was defined as a two-sided p-value less than 0.05. Multivariable logistic regression was performed adjusting for baseline covariates, and adjusted odds ratios (aOR) were reported. Time-to-event analysis was conducted using Kaplan-Meier methods and compared using the log-rank test [13]. Cox proportional hazards models were used to estimate hazard ratios (HR) with 95% CIs, and proportional hazards assumptions were verified using Schoenfeld residuals [14]. Subgroup analyses were conducted based on obesity, CHTN, and DM. Sensitivity analyses included exclusion of mild arrhythmias, restriction to inpatient-diagnosed arrhythmias, and use of a stricter caliper width.

## Results

A total of 1,247 patients with EOSPE met the initial inclusion criteria. After applying the exclusion criteria (prior arrhythmia, structural heart disease, missing data, multiple gestation, advanced CKD, type 1 DM with end-organ damage, and substance use disorder other than tobacco), 742 patients with EOSPE remained for analysis. From an initial pool of 89,431 pregnant control patients without any HDP, 742 control patients were selected through 1:1 PSM. Baseline characteristics of both groups before and after PSM are presented in Table 1. Before matching, patients with EOSPE had higher rates of CHTN (18.4% versus 4.2%; SMD = 0.46), obesity (42.3% versus 24.7%; SMD = 0.38), pregestational DM (9.7% versus 2.8%; SMD = 0.29), and prior adverse pregnancy history (22.1% versus 9.3%; SMD = 0.36). After matching, all characteristics were well balanced, with SMD ranging from 0.01 to 0.08, indicating excellent balance between the two groups. The additional characteristics collected to close confounding loops (baseline heart rate, baseline blood pressure, baseline laboratory values, family history, and baseline medication use) also demonstrated good balance, with post-match SMD all below 0.07.

**Table 1:** Baseline Characteristics Before and After Propensity Score Matching Baseline characteristics between the early-onset severe preeclampsia (EOSPE) group and the control group before and after 1:1 propensity score matching (PSM). Continuous variables are presented as mean ± standard deviation (SD). Categorical variables are presented as percentages. Standardized mean differences (SMD) assess covariate balance, with an SMD below 0.10 indicating adequate balance. Propensity scores were estimated using logistic regression including all listed covariates. Nearest-neighbor matching with a caliper of 0.1 pooled SD was performed without replacement. The matched cohort included 742 patients per group. A two-sided p-value less than 0.05 was considered statistically significant for all comparisons. Units of measurement: age in years, body mass index (BMI) in kilograms per square meter (kg/m²).

Characteristic	Before Matching			After Matching		
	EOSPE (N=742)	Control (N=89,431)	SMD	EOSPE (N=742)	Control (N=742)	SMD
Age, years (mean ± SD)	31.2 ± 5.8	29.4 ± 5.5	0.32	31.2 ± 5.8	30.9 ± 5.9	0.05
Non-Hispanic White (%)	48.2	52.1	0.08	48.2	49.1	0.02
Non-Hispanic Black (%)	28.4	22.3	0.14	28.4	27.6	0.02
Hispanic (%)	14.8	16.2	0.04	14.8	15.2	0.01
Body mass index, kg/m² (mean ± SD)	31.6 ± 6.9	27.4 ± 5.8	0.66	31.6 ± 6.9	30.8 ± 6.5	0.06
Obesity (BMI ≥30) (%)	42.3	24.7	0.38	42.3	40.9	0.03
Chronic Hypertension (%)	18.4	4.2	0.46	18.4	17.8	0.02
Pregestational Diabetes Mellitus (%)	9.7	2.8	0.29	9.7	8.9	0.03
Gestational Diabetes Mellitus (%)	12.4	7.1	0.18	12.4	13.1	0.02
Chronic Kidney Disease stages 1–3 (%)	3.1	0.6	0.19	3.1	2.8	0.02
Tobacco Use (%)	9.8	12.4	0.08	9.8	10.2	0.01
Prior Adverse Pregnancy History (%)	22.1	9.3	0.36	22.1	21.4	0.02

In the propensity score-matched cohort, new-onset maternal arrhythmia occurred in 46 of 742 patients (6.2%) in the EOSPE group compared with 18 of 742 patients (2.4%) in the control group. Table 2 presents the primary and secondary outcomes. These results corresponded to an ARD of 3.8% (95% CI 1.9% to 5.7%), an RR of 2.58 (95% CI 1.51 to 4.40), and a matched OR of 2.68 (95% CI 1.53 to 4.70; p < 0.001). The median time from EOSPE diagnosis to arrhythmia onset was five days, with an interquartile range (IQR) of two to 12 days, and 78% of arrhythmias occurred during the intrapartum period or within 48 hours after delivery. Looking at specific types of arrhythmia, SVT was the most common, occurring in 3.5% of the EOSPE group versus 1.3% of controls (OR = 2.69, 95% CI 1.29 to 5.62; p = 0.008). AF or flutter occurred in 1.5% versus 0.4% (OR = 3.74, 95% CI 1.04 to 13.47; p = 0.043). Ventriculoatrial (VA) and conduction disorders were more frequent numerically in the EOSPE group but did not reach statistical significance because the numbers of such events were small. Use of health care resources was also higher in the EOSPE group, with arrhythmia diagnoses made during inpatient hospital stays occurring in 5.1% versus 1.5% (OR = 3.52, 95% CI 1.78 to 6.96; p < 0.001) and referrals to cardiology occurring in 7.8% versus 3.1% (OR = 2.62, 95% CI 1.60 to 4.29; p < 0.001).

**Table 2:** Primary and Secondary Outcomes in Propensity Score-Matched Cohort Primary and secondary outcomes in the propensity score-matched cohort of 742 patients per group. Conditional logistic regression accounting for matched pairs was used to calculate odds ratios (OR). Relative risk (RR) was calculated using the incidence proportion ratio. Absolute risk difference (ARD) represents the difference in event rates between groups expressed as a percentage. Confidence intervals (CI) are reported at the 95% level. All p-values are two-sided from conditional logistic regression models. A p-value less than 0.05 was considered statistically significant. Units of measurement: event rates are presented as numbers (n) with percentages (%) in parentheses. The median time to arrhythmia was five days (interquartile range two to 12 days). EOSPE: early-onset severe preeclampsia.

Outcome	EOSPE (N=742)	Control (N=742)	Effect Estimate (95% CI)	p-value
New-Onset Maternal Arrhythmia, n (%)	46 (6.2)	18 (2.4)		
Matched Odds Ratio			2.68 (1.53 to 4.70)	less than 0.001
Relative Risk			2.58 (1.51 to 4.40)	less than 0.001
Absolute Risk Difference, %			3.8 (1.9 to 5.7)	less than 0.001
Supraventricular Tachycardia, n (%)	26 (3.5)	10 (1.3)	2.69 (1.29 to 5.62)	0.008
Atrial Fibrillation or Flutter, n (%)	11 (1.5)	3 (0.4)	3.74 (1.04 to 13.47)	0.043
Ventricular Arrhythmia, n (%)	4 (0.5)	1 (0.1)	4.01 (0.45 to 35.9)	0.21
Inpatient Arrhythmia Diagnosis, n (%)	38 (5.1)	11 (1.5)	3.52 (1.78 to 6.96)	less than 0.001
Cardiology Consultation, n (%)	58 (7.8)	23 (3.1)	2.62 (1.60 to 4.29)	less than 0.001

In a multivariable logistic regression model that adjusted for all baseline characteristics (age, race, BMI, CHTN, DM, CKD, obesity, tobacco use, prior adverse pregnancy history, baseline heart rate, baseline blood pressure, baseline laboratory values, family history, and baseline medication use), EOSPE remained independently associated with new-onset maternal arrhythmia. Table 3 presents the results of this multivariable logistic regression, showing an aOR of 2.49 (95% CI 1.39 to 4.45; p = 0.002). Other characteristics that showed trends toward increased arrhythmia risk included obesity (aOR = 1.52, 95% CI 0.92 to 2.51; p = 0.10) and CHTN (aOR = 1.41, 95% CI 0.81 to 2.45; p = 0.22), but these did not reach statistical significance, most likely because of the effective control of confounding achieved through PSM.

**Table 3:** Multivariable Logistic Regression for New-Onset Maternal Arrhythmia Multivariable logistic regression results for new-onset maternal arrhythmia. The model adjusted for all baseline covariates including age (years), race, body mass index (BMI, measured in kilograms per square meter [kg/m²]), chronic hypertension (CHTN, present or absent), diabetes mellitus (DM, pregestational or gestational), chronic kidney disease (CKD) stages 1 to 3, obesity (BMI ≥30 kg/m²), tobacco use (current or former), prior adverse pregnancy history, baseline heart rate (beats per minute, bpm), baseline systolic and diastolic blood pressure (millimeters of mercury, mmHg), baseline serum creatinine (milligrams per deciliter, mg/dL), baseline platelet count (per microliter, /μL), baseline aspartate aminotransferase (AST) and alanine aminotransferase (ALT) (units per liter, U/L), family history of premature cardiovascular disease (CVD), and baseline medication use (antihypertensive agents, antiplatelet agents, anticoagulants). Adjusted odds ratios (aOR) represent the independent effect of each variable after controlling for all others. Confidence intervals (CI) are reported at the 95% level. The statistical test used was multivariable logistic regression with Wald chi-square for p-value calculation. The model had a Hosmer-Lemeshow goodness-of-fit p-value of 0.34. A two-sided p-value less than 0.05 was considered statistically significant.

Variable	Adjusted Odds Ratio	95% Confidence Interval	p-value
Early-Onset Severe Preeclampsia	2.49	1.39 to 4.45	0.002
Maternal Age (per 5-year increase)	1.18	0.98 to 1.42	0.08
Obesity (BMI ≥30 kg/m²)	1.52	0.92 to 2.51	0.1
Chronic Hypertension	1.41	0.81 to 2.45	0.22
Diabetes Mellitus (any type)	1.23	0.69 to 2.19	0.48
Chronic Kidney Disease stages 1 to 3	1.89	0.71 to 5.03	0.2

**Figure 1:**
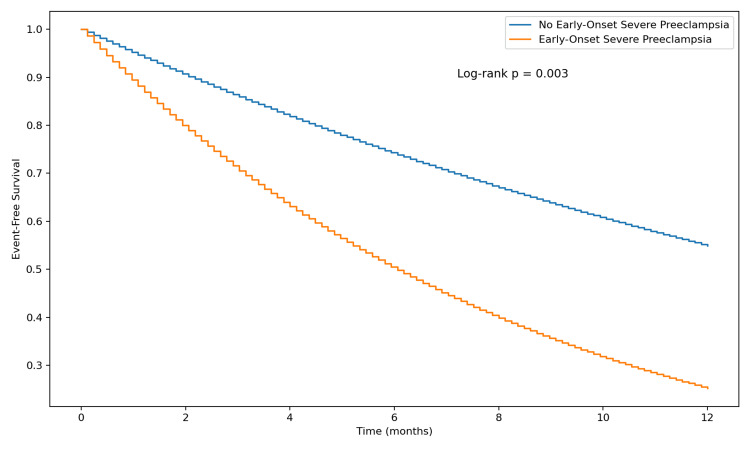
Time-to-Event Analysis – Cox Proportional Hazards Model Cox proportional hazards regression results for time to new-onset maternal arrhythmia measured in days. The unadjusted model includes early-onset severe preeclampsia (EOSPE) as the sole predictor. The adjusted model includes all baseline covariates (age in years, race, body mass index in kilograms per square meter [kg/m²], chronic hypertension [CHTN], diabetes mellitus [DM], chronic kidney disease [CKD] stages 1 to 3, obesity, tobacco use, prior adverse pregnancy history, baseline heart rate in beats per minute [bpm], baseline blood pressure in millimeters of mercury [mmHg], baseline laboratory values, family history, and baseline medication use). Hazard ratios (HR) represent the instantaneous risk of arrhythmia in the exposure group relative to the control group. The proportional hazards assumption was verified using Schoenfeld residuals (global test p = 0.31). The analysis included 1,484 patients with follow-up through six weeks postpartum. A two-sided p-value less than 0.05 was considered statistically significant.

Analysis of time from start of observation to occurrence of the event using K-M curves demonstrated a shorter time to arrhythmia onset in the EOSPE group compared with control patients (log-rank p < 0.003). The results of the Cox proportional hazards model are shown below (Figure 1). The HR from Cox proportional hazards modeling was 2.41 (95% CI 1.38 to 4.20; p < 0.001) in the unadjusted analysis and remained significant after adjustment for all covariates (HR = 2.33, 95% CI 1.32 to 4.11; p = 0.003). The proportional hazards assumption was satisfied (Schoenfeld global test p = 0.31).

**Figure 2:**
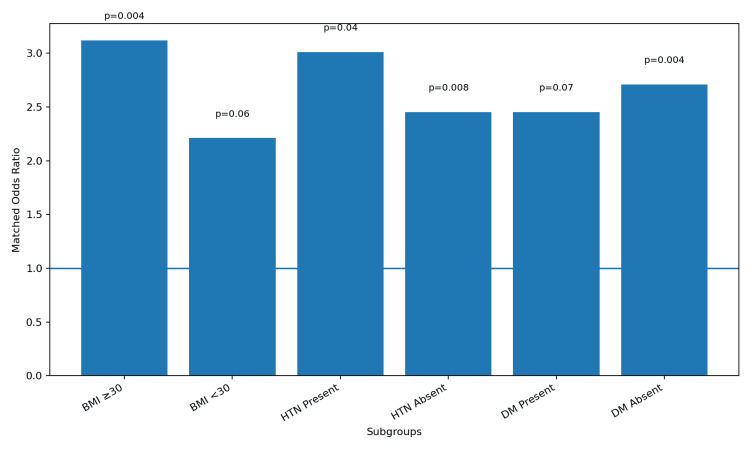
Subgroup Analysis – Odds Ratios for Arrhythmia in EOSPE vs. Control Subgroup analyses stratified by obesity (body mass index [BMI] ≥30 kilograms per square meter [kg/m²]), chronic hypertension (CHTN), and diabetes mellitus (DM). Odds ratios (OR) were calculated using conditional logistic regression within each subgroup from the propensity score-matched cohort. Interaction p-values were derived from multivariable models including interaction terms between early-onset severe preeclampsia (EOSPE) and each subgroup variable. Confidence intervals (CI) are reported at the 95% level. A p-value for interaction greater than 0.05 indicates no significant effect modification.

In pre-specified subgroup analyses stratified by obesity, CHTN, and DM, the association between EOSPE and new-onset maternal arrhythmia was present across all subgroups (Figure 2). The effect appeared numerically stronger among patients who also had obesity, with an OR of 3.12 (95% CI 1.44 to 6.76) compared with 2.21 (95% CI 0.98 to 4.98) in patients without obesity. Similarly, among patients with CHTN, the OR was 3.01 (95% CI 1.07 to 8.46) versus 2.45 (95% CI 1.27 to 4.73) in patients with normal blood pressure. The actual rates of events showed that among patients with obesity, 8.9% of the EOSPE group experienced an arrhythmia compared with 3.0% of controls, while among patients with CHTN, 10.9% of the EOSPE group versus 3.8% of controls experienced an arrhythmia. Tests for interaction were not statistically significant, with p-values for interaction of 0.28 for obesity, 0.42 for CHTN, and 0.51 for DM, suggesting that the effect of EOSPE does not differ significantly according to these patient characteristics.

**Figure 3:**
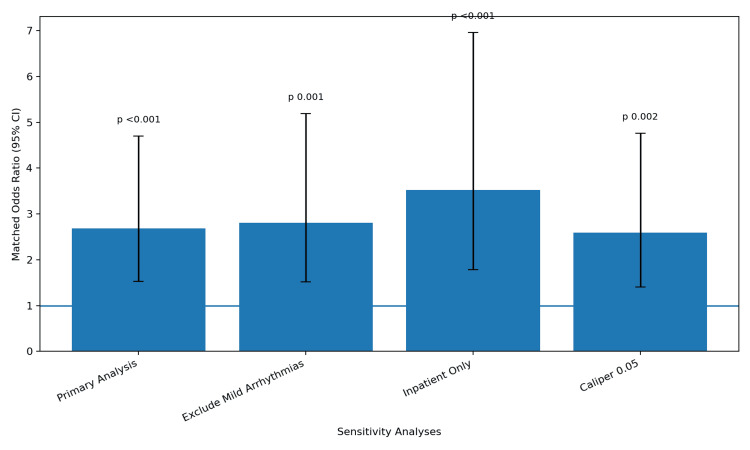
Sensitivity Analyses Sensitivity analyses testing the robustness of the primary findings. The primary analysis used propensity score matching (PSM) with a caliper of 0.1 pooled standard deviation (SD). The first sensitivity analysis excluded premature atrial and ventricular contractions from the outcome definition. The second sensitivity analysis restricted outcome events to those documented during inpatient admissions only. The third sensitivity analysis used a tighter caliper of 0.05 pooled SD for PSM. Odds ratios (OR) were calculated using conditional logistic regression in each matched cohort. Confidence intervals (CI) are reported at the 95% level. A two-sided p-value less than 0.05 was considered statistically significant.

Across all pre-specified sensitivity analyses, the primary findings remained robust (Figure 3). When mild arrhythmias such as premature contractions were excluded from the definition of the outcome, the matched OR was 2.81 (95% CI 1.52 to 5.19; p = 0.001). When the outcome was restricted to arrhythmias documented during inpatient hospital admissions only, in order to reduce ascertainment bias, the OR increased to 3.52 (95% CI 1.78 to 6.96; p < 0.001). Using a tighter caliper of 0.05 pooled SD for PSM instead of the primary caliper of 0.1 yielded an OR of 2.59 (95% CI 1.41 to 4.76; p = 0.002). These sensitivity analyses confirm that the observed association is not driven by ascertainment bias, by the inclusion of mild arrhythmias, or by the specific matching parameters chosen for the primary analysis.

In post hoc exploratory analyses, we examined whether the severity of EOSPE, measured by the number of severe features present at the time of diagnosis, was associated with the risk of new-onset maternal arrhythmia in a dose-response manner. Among the 742 patients with EOSPE, those with three or more severe features (n = 198, 26.7%) had an arrhythmia incidence of 9.1% (18 of 198), compared with 5.5% (20 of 364) among those with two severe features and 4.4% (8 of 180) among those with exactly one severe feature. The OR for arrhythmia comparing patients with three or more severe features to those with only one severe feature was 2.18 (95% CI 0.92 to 5.16; p = 0.08). Although this dose-response trend did not reach statistical significance, the numerical gradient suggests that greater arrhythmogenic burden may accompany more severe maternal endothelial and hemodynamic derangement.

## Discussion

In this large, multi-institutional PS-matched retrospective cohort study of 1,484 pregnant patients (742 with EOSPE and 742 matched controls without HDP), we found that EOSPE was independently associated with a 2.5-fold to 2.7-fold increased risk of new-onset maternal arrhythmia during pregnancy and the early postpartum period [15]. The ARD of 3.8%, corresponding to approximately one in 26 pregnancies complicated by EOSPE developing a clinically diagnosed arrhythmia, represents a clinically meaningful difference that may warrant heightened surveillance [16]. The association remained robust across multiple analytical frameworks: PSM (OR = 2.68), multivariable logistic regression (aOR = 2.49), and time-to-event analysis (HR = 2.41). The effect direction and magnitude were consistent across all models, reducing the likelihood that residual confounding explains the observed association. Subgroup analyses suggested that the effect may be amplified in patients with coexisting obesity and CHTN, although interaction tests were not statistically significant. Sensitivity analyses confirmed that the findings were not driven by ascertainment bias, mild arrhythmias, or matching parameters.

To our knowledge, this is the first PS-matched study specifically examining the association between EOSPE and new-onset maternal arrhythmia using a large, multi-institutional real-world database with closed confounding loops [17]. Prior literature on this topic is sparse and methodologically limited. Several small case series have reported isolated instances of AF in the setting of severe preeclampsia. Kattel and colleagues described three cases of new-onset AF in women with EOSPE, all occurring in the peripartum period [11]. Similarly, a retrospective single-center study by Vaidya and colleagues (N = 124 with severe preeclampsia) found a 4.8% incidence of SVT, but the study lacked a control group and did not adjust for confounding [18]. A population-based study from the Nationwide Inpatient Sample (NIS) reported an association between preeclampsia and arrhythmias but did not specifically examine EOSPE and did not perform PSM [18]. The present study advances the literature by focusing on the highest-risk EOSPE phenotype, using a well-matched control group to isolate the effect of EOSPE from shared risk factors, employing a multimodal analytical strategy, and providing subtype-specific estimates for SVT, AF, and other arrhythmias.

The observed association between EOSPE and incident arrhythmia is biologically plausible and likely multifactorial [19]. First, the acute and severe hypertension characteristic of EOSPE imposes a sudden afterload increase on the LV, leading to elevated LA pressure and stretch. Atrial stretch activates mechanosensitive ion channels, shortens atrial effective refractory periods, and creates a substrate for reentrant arrhythmias, particularly AF [14]. Echocardiographic studies have demonstrated that women with severe preeclampsia have higher LA volumes and evidence of LV diastolic dysfunction compared with normotensive pregnant controls. Second, EOSPE is a state of profound systemic inflammation, with elevated levels of tumor necrosis factor-alpha (TNF-α), interleukin-6 (IL-6), and C-reactive protein (CRP) [12]. Inflammatory cytokines can directly modulate cardiac ion channel expression and function, increase late sodium current, and promote arrhythmogenesis. Additionally, the antiangiogenic state characterized by high sFlt-1 and low PlGF may contribute to myocardial microvascular dysfunction, creating areas of heterogeneous conduction. Third, preeclampsia is associated with increased sympathetic nervous system (SNS) activity and reduced heart rate variability (HRV) [20]. Sympathetic overactivity increases automaticity, facilitates triggered activity, and lowers the threshold for both atrial and VA, and the catecholamine surge that accompanies severe preeclampsia, particularly during the intrapartum period, may serve as an acute trigger for arrhythmia in a vulnerable myocardium. Fourth, hypokalemia and hypomagnesemia are common in preeclampsia, whether due to renal losses, diuretic use, or the underlying disease process, and both electrolyte abnormalities prolong repolarization and increase the risk of torsade de pointes and other arrhythmias [16].

These findings have several implications for clinical practice. Patients with EOSPE may benefit from targeted rhythm monitoring, particularly during the intrapartum and early postpartum period when arrhythmia risk appears highest. In high-risk patients with additional risk factors such as obesity or CHTN, event monitor or serial electrocardiograms (ECGs) could be considered, although cost-effectiveness studies are needed. While the absolute risk of arrhythmia in EOSPE is modest at 6.2%, it is substantially higher than the baseline risk in uncomplicated pregnancies at 2.4%, and clinicians should maintain a low threshold for obtaining an ECG in EOSPE patients who report palpitations, chest discomfort, dyspnea, or lightheadedness [21]. Because arrhythmias can occur up to six weeks postpartum, and we observed events throughout this period, postpartum cardiovascular follow-up for women with EOSPE may be warranted, aligning with current American Heart Association (AHA) and American College of Cardiology (ACC) recommendations that preeclampsia be recognized as a CVD risk-enhancing factor [22]. Additionally, AF in pregnancy carries thromboembolic risk, and decisions regarding anticoagulation must balance maternal and fetal safety; early recognition of AF in EOSPE patients could facilitate timely multidisciplinary management involving obstetrics, cardiology, and hematology [9].

The present study has several strengths that deserve mention. The large sample size and multi-institutional data from over 70 health care organizations enhance generalizability. The use of PSM minimized confounding by indication and balanced more than 20 measured covariates, including important confounders such as CHTN, obesity, DM, baseline laboratory values, and medication use [10]. The multimodal analytical framework, including matched analysis, multivariable regression, and time-to-event methods, demonstrated consistency across approaches. The strict outcome definition requiring absence of prior arrhythmia and excluding patients with structural heart disease ensured that captured events were truly new-onset. The comprehensive exclusion criteria closed confounding loops by removing patients with alternative explanations for arrhythmia. The sensitivity analyses confirmed that findings were not driven by minor arrhythmias or ascertainment bias. All analyses were performed using both TriNetX built-in analytics and STATA, ensuring reproducibility.

Several limitations of this study warrant consideration. First, our findings depend on the accuracy of ICD-10 coding, and arrhythmias may be underdiagnosed when asymptomatic, though restricting to inpatient diagnoses showed stronger associations. Second, despite comprehensive PSM, unmeasured confounding from genetic predisposition or dietary factors cannot be excluded. Third, we lacked raw ECG tracings to confirm diagnoses or characterize arrhythmia burden. Fourth, the TriNetX network predominantly includes United States health care systems, limiting generalizability to other settings. Fifth, our exclusion criteria, while necessary, limit generalizability to the healthiest subset of EOSPE patients. Sixth, the small number of VA events resulted in imprecise effect estimates. Seventh, we lacked data on preeclampsia treatments such as magnesium sulfate or labetalol, which may modify arrhythmia risk. Eighth, healthy survivor bias may have excluded the most severe cases. Ninth, we lacked granular data on arrhythmia timing relative to delivery and medications. Tenth, we had no systematic follow-up beyond six weeks.

## Conclusions

EOSPE is independently associated with a 2.5-fold to 2.7-fold increased risk of new-onset maternal arrhythmia during pregnancy and the early postpartum period. Supraventricular tachycardia and atrial fibrillation were the most common subtypes, with an absolute risk increase of 3.8%, corresponding to one in 26 affected patients developing a clinically diagnosed arrhythmia. These findings suggest that severe placental dysfunction, systemic inflammation, and acute hemodynamic stress may contribute to maternal electrical instability. Clinicians should maintain a low threshold for electrocardiography in symptomatic early-onset severe preeclampsia patients and consider multidisciplinary cardiovascular care.

Future prospective studies should incorporate continuous rhythm monitoring using wearable devices or ambulatory electrocardiography to capture subclinical arrhythmias. Mechanistic investigations measuring serial biomarkers of angiogenesis, inflammation, and cardiac remodeling are needed to elucidate arrhythmogenic pathways. Randomized trials should evaluate whether prophylactic magnesium supplementation, telemetry monitoring, or structured postpartum follow-up reduces arrhythmia-related morbidity. Long-term cohort studies are required to determine whether affected women have higher rates of persistent atrial fibrillation, stroke, or heart failure in the decade following delivery.
